# Ferrocene Derivatives for Improving the Efficiency and Stability of MA‐Free Perovskite Solar Cells from the Perspective of Inhibiting Ion Migration and Releasing Film Stress

**DOI:** 10.1002/advs.202304790

**Published:** 2023-10-22

**Authors:** Huan Bi, Jiaqi Liu, Zheng Zhang, Liang Wang, Gaurav Kapil, Yuyao Wei, Ajay Kumar Baranwal, Shahrir Razey Sahamir, Yoshitaka Sanehira, Dandan Wang, Yongge Yang, Takeshi Kitamura, Raminta Beresneviciute, Saulius Grigalevicius, Qing Shen, Shuzi Hayase

**Affiliations:** ^1^ i‐Powered Energy System Research Center (i‐PERC) The University of Electro‐Communications 1‐5‐1 Chofugaoka, Chofu Tokyo 182‐8585 Japan; ^2^ Faculty of Informatics and Engineering The University of Electro‐Communications 1‐5‐1 Chofugaoka, Chofu Tokyo 182‐8585 Japan; ^3^ Department of Polymers Chemistry and Technology Kaunas University of Technology Radvilenu Plentas 19 Kaunas LT50254 Lithuania

**Keywords:** ferrocene derivatives, ion migration, MA‐free perovskite solar cells, monomolecular layer, stress relief

## Abstract

Further improvement of the performance and stability of inverted perovskite solar cells (PSCs) is necessary for commercialization. Here, ferrocene derivative dibenzoylferrocene (DBzFe) is used as an additive to enhance the performance and stability of MA‐ and Br‐ free PSCs. The results show that the introduction of DBzFe not only passivates the defects in the film but also inhibits the ion migration in the film. The final device achieves a power conversion efficiency (PCE) of 23.53%, which is one of the highest efficiencies currently based on self‐assembled monolayers (SAMs). Moreover, it maintains more than 96.4% of the original efficiency when running continuously for 400 h at the maximum power point.

## Graphical abstract

Using a new ferrocene derivative (DBzFe) as an additive, the defect of the film is significantly reduced due to the chemical interaction between DBzFe and perovskite. Meanwhile, the introduction of additives further hinders ion migration in the film and releases harmful stress in the film. Ultimately, the photovoltaic performance and long‐term stability of the device are significantly improved.

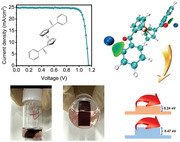

## 1 Introduction

Perovskite solar cells (PSCs) have become the mainstay of the third‐generation photovoltaic market due to their long carrier lifetime, tunable bandgap, and easy preparation by low‐temperature solution method.^[^ ^1^, ^2^, ^3^, ^4^ ^]^ Currently, the certified efficiency of single‐cell perovskite solar cells has reached 26.0%, comparable to silicon‐based solar cells.^[^ ^5^ ^]^ However, according to the Shockley–Queisser limit,^[^ ^6^ ^]^ the power conversion efficiency (PCE) of PSCs still has a lot of room for improvement. As reported, poor film quality and severe nonradiative recombination can all reduce the device's efficiency.^[^ ^7^, ^8^, ^9^, ^10^ ^]^ Therefore, further improving perovskite film quality and reducing device nonradiative recombination is critical to improving device efficiency. Currently, most high‐efficiency PSCs with PCEs exceeding 23% are fabricated using methylammonium (MA)‐containing perovskite compositions, such as MAFA‐based and CsMAFA‐based perovskites.^[^ ^9^, ^11^, ^12^, ^13^, ^14^ ^]^ However, it has been observed that MA^+^ tends to volatilize and escape from the perovskite crystal lattice, resulting in poor thermal stability. Even at relatively low temperatures, ≈80 °C, decomposition of MAPbI_3_ occurs.^[^ ^15^, ^16^ ^]^ Furthermore, MAPbI_3_ can convert from a tetragonal to a cubic phase at 55 °C, negatively impacting device stability. In addition, MAPbI_3_ is susceptible to degradation under light and moisture exposure, further reducing its stability.^[^ ^17^, ^18^ ^]^ In summary, the instability caused by poor thermal, phase, light, and moisture stability presents a significant challenge for the commercial application of MA‐containing PSCs. Consequently, exploring the development of MA‐free PSCs as a feasible and effective approach to address these instability issues in the MA‐containing counterparts becomes essential.^[^ ^19^, ^20^ ^]^

Improving the quality of perovskite films is considered a meaningful way to enhance the device's efficiency and stability.^[^ ^21^, ^22^, ^23^, ^24^ ^]^ The additive strategy has been proven effective in improving the quality of thin films and the efficiency of devices.^[^ ^8^, ^25^, ^26^ ^]^ Chen and his co‐worker developed a precursor stabilization and defect passivation strategy by employing 3‐hydrazinobenzoic acid (3‐HBA) containing both carboxyl (−COOH) and hydrazine (−NHNH_2_) functional groups as a versatile additive. The results show that the introduction of 3‐HBA improves the quality of the perovskite film while suppressing the nonradiative recombination in the device. As a result, an efficiency of 23.3% with a perovskite composition of FA_0.95_Cs_0.05_PbI_3_ was obtained.^[^ ^27^ ^]^ Park's group report on the dependence of photovoltaic performance and stability of PSCs on the additive of primary alkylammonium chloride with different alkyl chains. After a screening experiment, an in‐depth study is performed with propylammonium chloride (PACl) because PACl shows the best photovoltaic performance among the studied candidates. The 20 mol% PACl to FAPbI_3_ leads to enhanced grain size, crystal growth with a preferred orientation, effective passivation of defects, and reduced Urbach energy, which results in a PCE of 22.22%.^[^ ^28^ ^]^ Introducing additives has also proven effective in relieving the film's stress and inhibiting the film's ion migration. Yi and his co‐authors developed a novel multifunctional organic salt, methylammonium succinate, which can alleviate strain and reinforce grain boundaries, which was incorporated into the perovskite film, leading to a relaxed micro‐strain and a lower defect concentration. As a result, a certified PCE of 22.5% for FAPbI_3_ was achieved.^[^ ^29^ ^]^ Lee also used acetamidinium bromide (AABr) as the additive, thereby releasing the film's stress and finally improving the device's performance.^[^ ^30^ ^]^

This work used a ferrocene derivative named dibenzoylferrocene (DBzFe) as an additive to prepare efficient and stable MA‐free perovskite solar cells. We found that DBzFe can passivate perovskite defects, improve film quality, and inhibit ion migration in the film. Both experiments and theoretical calculations reveal a strong chemical interaction between DBzFe and perovskite. Given this, the corresponding DBzFe‐based device exhibits a PCE as high as 23.53%, which is the highest efficiency based on self‐assembled monolayers (SAMs). In addition, the target device also exhibited excellent long‐term stability, including light stability, and thermal stability.

## 2 Results and Discussion

**Figure 1:**
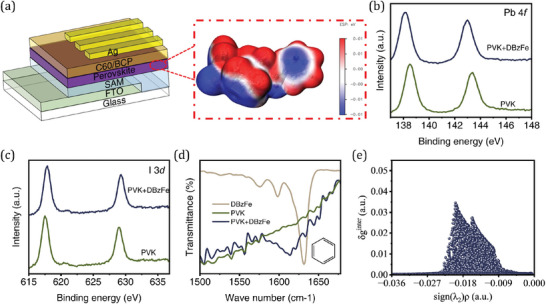
a) Device structure of the PSCs used in this work and the electrostatic potential of DBzFe. XPS b) Pb 4*f* and c) I 3*d* of the perovskite film with or without DBzFe modification. d) FTIR spectra of DBzFe, perovskite, and perovskite+DBzFe films. e) Scatter map between δ_g_^inter^ and sign(λ_2_)ρ.

The device structure used in this work is shown in **Figure** **1**a, FTO/SAMs/perovskite/C60/BCP/Ag, where the MA‐ and Br‐free perovskite composition by Cs_0.05_FA_0.95_PbI_3_ was employed to fabricate light absorbing layer. The DBzFe was used as the additive, and the structure is shown in Figure S1 (Supporting Information). DBzFe has a symmetrical multifunctional structure, and the electrostatic potential (ESP) map of DBzFe is depicted in Figure 1a, –C=O is expected to form a chemical interaction with undercoordinated Pb^2+^ in the perovskite film to reduce defects in the film due to its lone pair of electrons. Meanwhile, benzene rings have also been widely reported to inhibit the migration of I^−^ in perovskite thin films.^[^ ^31^, ^32^, ^33^, ^34^, ^35^ ^]^

X‐ray photoelectron spectroscopy (XPS) was used to uncover the chemical interaction between perovskite and DBzFe. As shown in Figure 1b, the perovskite film with DBzFe modified (143.0 and 138.1 eV) exhibited a Pb 4*f* peak shift compared with pristine perovskite film 143.4 and 138.5 eV). In addition, the shift of I 3*d* peaks in perovskite with (617.9 and 629.3 eV) or without (617.5 and 629.0 eV) DBzFe modification certified the chemical interaction between DBzFe and perovskites (Figure 1c). Subsequently, to investigate the interaction between Fe (from DBzFe) and perovskite, the results are presented in Figure S2 (Supporting Information). When DBzFe is introduced into the perovskite, the Fe 2*p* peak shifts to a lower binding energy. This shift indicates that Fe from DBzFe can form a chemical interaction with the perovskite, potentially enhancing the quality of the film. Fourier‐transform infrared spectroscopy (FTIR) was also used to demonstrate the chemical interaction between DBzFe and perovskites. As shown in Figure 1d, the peak located at 1614.1 cm^−1^ was assigned to the vibration of the benzene ring.^[^ ^36^, ^37^ ^]^ Here, for the perovskite film, no obvious peak position is observed, while for perovskite+DBzFe film, a distinct benzene ring vibration peak can be observed, which suggests that the DBzFe indeed introduced into the perovskite thin film. Compared with DBzFe powder, the peak position of the benzene ring belonging to perovskite+DBzFe film shifted, indicating a weak interaction between perovskite and the benzene ring. DFT calculations with an independent gradient model based on the Hirshfeld partition of molecular density method help clarify the weak interactions between perovskite and DBzFe (Figure 1e; Figure S2, Supporting Information).^[^ ^38^ ^]^ It can be observed that two obvious peaks are located between −0.036 and 0.000 a.u., indicating weak interactions in the system. Figure S3 (Supporting Information) further shows this support‐weak interaction. It can be seen that there is a green large‐area flat sheet between I and the benzene ring, which visually proves that there is an interaction between I and the benzene ring (the chromaticity card gives the color and value range of each force) and is consistent with paper reports.^[^ ^39^, ^40^ ^]^ In summary, we have demonstrated experimentally and computationally that a chemical interaction exists between the perovskite film and DBzFe.

**Figure 2:**
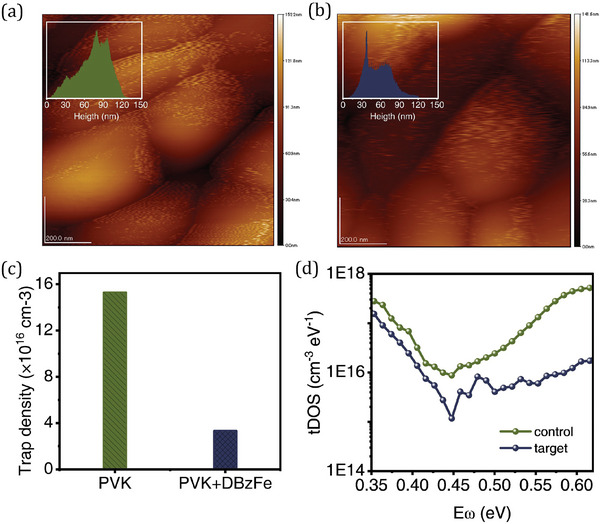
AFM of the perovskite film a) without and b) with DBzFe modification. c) Trap density of the perovskite film with or without DBzFe modification calculated from TIPL result. d) *t*DOS of the two types of devices.

UV–vis is used to study the optical properties of perovskite thin films. As shown in Figure S4a (Supporting Information), compared with the control film, the target film showed higher absorbance intensity. At the same time, the bandgap of the perovskite film calculated from the Tauc plot is 1.52 eV for the control film and the target film (Figure S4b, Supporting Information). Atomic force microscopy (AFM) was used to study the perovskite film's roughness with or without DBzFe modification. **Figure** **2**a,b shows that the target perovskite film exhibits a flatter surface than the control perovskite film. We evaluated the film roughness (Rq) of the observed area and showed that the target film (Rq = 22.1 nm) indeed exhibited less roughness compared to the control film (Rq = 26.5 nm). The inset shows the grain roughness statistics in the area. At the same time, the target film exhibited a tighter grain arrangement. Scanning electron microscopy (SEM) measurement was also carried out to study the effect of the DBzFe treatment on the morphology of perovskite films. As shown in Figure S5 (Supporting Information), compared with the control device, the grain size of the target film is significantly increased, which may be attributed to the chemical interaction between DBzFe and perovskite, which affects the crystallization process of the perovskite film. In addition, according to the photoacoustic spectroscopy (PA, Figure S6, Supporting Information), perovskite with DBzFe modification has a steeper band tail with a lower Urbach energy of 37.9 meV, which is lower than that of the control film (58.7 meV). This result implies that the DBzFe substantially impacts the electronic band tails of the perovskite films, lowering trap density and electronic disorder.^[^ ^41^, ^42^ ^]^ We investigated the trap density of states for control and target perovskite films by analyzing the dependency of the time‐integrated PL intensity (TIPL) on the pumping flux.^[^ ^41^ ^]^ As shown in Figure S7 (Supporting Information), Figure 2c, and Note S1 (Supporting Information), the fitted trap density of the target perovskite film is 3.32 × 10^16^ cm^−3^, which is lower than that of the control film (15.28 × 10^16^ cm^−3^), indicating that DBzFe can passivate the defects in perovskite films to minimize competition for nonradiative recombination. PL and TRPL were also used to uncover the defect of the perovskite with or without DBzFe added. As shown in Figure S8 (Supporting Information), for the PL result, after intruding the DBzFe into the perovskite solution, the intensity of the perovskite film is higher than that without DBzFe modification. In the meantime, the TRPL also suggests that the DBzFe can improve the quality of the perovskite film. Hall effect was also carried out to uncover the effect of the DBzFe on perovskite film mobility. As shown in Table S2 (Supporting Information), after DBzFe was introduced, the mobility of the perovskite film increased. *C‐f* measurements were conducted to analyze the trap density of states (tDOS) in energy space for the control and target devices to investigate it further.^[^ ^43^ ^]^ Figure 2d shows the tDOS curves of the two devices, in which the value of Eω >0.4 eV is defined as the deep‐level defect of the film. It can be seen that the target film exhibits a lower defect density compared to the pristine film. This result suggests that DBzFe can effectively passivate the deep‐level defects of perovskite films.^[^ ^44^, ^45^ ^]^

**Figure 3:**
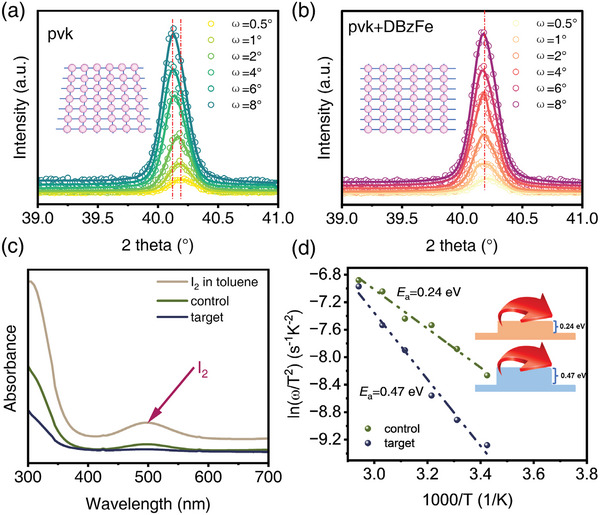
GIXRD patterns with different ω values (0.5°–8°) for a) control and b) DBzFe‐modified perovskite films. c) Ultraviolet absorption spectra of toluene solutions soaked with perovskite films with or W/O DBzFe modification under 1 sun illumination. d) Ion migration activation energy assessment by using temperature‐dependent conductivity measurements.

As reported, the film stress can accelerate perovskite film breakdown and device failure.^[^ ^20^, ^46^ ^]^ Here, we performed (GIXRD) measurements to analyze the residual stress of the control and modified perovskite films.^[^ ^20^, ^46^, ^47^, ^48^ ^]^ A series of slight incidence angles (ω from 0.5° to 8°) are selected to explore the information of perovskite thin films at different depths. With the increase of the angle of incidence, the information on the deeper perovskite film can be captured. As shown in Figures 3a and b, for the control film (**Figure** **3**a), with the angle of incidence increases, the diffraction angle 2θ gradually shifts to lower angles, according to the Bragg law (λ = 2*d*sinθ), which means that as the film depth increases, the lattice spacing of the film changes, resulting in stresses inside the film and be classified to tensile stress. While for the target film, as the detection angle increases, the diffraction angle of the film hardly shifts, which means that the addition of DBzFe releases the stress in the film, and the reduced defects by DBzFe and flexible DBzFe can be responsible for mitigating stress. Strain in perovskite films has been reported to significantly impact the material's band structure, carrier mobility, ion migration, and defect formation energy.^[^ ^49^ ^]^ Chen and co‐authors report that large tensile strains increase the bandgap, while compressive stresses can reduce the bandgap of the film.^[^ ^47^ ^]^ They also found that by adjusting the stacking mode of A‐site cations, the micro‐strain of the film can be relieved, thereby improving the carrier mobility of the film.^[^ ^50^ ^]^ In addition, some reports claimed that compressive strain can also increase the activation energy of ion migration, whereas tensile stress decreases the activation energy of ion migration (we discussed in the next step).^[^ ^51^ ^]^ In a word, the released micro‐strain reduces the nonradiative recombination of carriers, which is beneficial for us further to improve the PCE and stability of the device.

Next, we evaluated the effect of DBzFe on ion migration in perovskite thin films. It has been reported that undercoordinated I ions or I vacancy defects in perovskite films can aggravate ion migration and lead to an irreversible decline in device performance.^[^ ^52^, ^53^, ^54^ ^]^ Huang et al. observed obvious I ion suppression by additive using UV–vis by immersing perovskite films in a toluene solution. This is because toluene can effectively dissolve I_2_ without destroying the perovskite film.^[^ ^55^, ^56^ ^]^ This similarly motivates us to evaluate ion transport in thin films. We used the setup of Figure S9a (Supporting Information) to evaluate the degree of ion migration of perovskite thin films under 1 sun illumination. After aging for 24 h, the control film (Figure S9b, Supporting Information) shows significant degradation, while the target perovskite film (Figure S9c, Supporting Information) still maintains good coverage. This result indicates that the introduction of DBzFe is beneficial in improving the stability of the film under light conditions by inhibiting ion migration. At the same time, UV–vis was used to study the effect of DBzFe on ion migration in the perovskite film following the report. We first characterize the UV–vis signal of I_2_ dissolved in toluene. As shown in Figure 3c, there is an obvious absorption peak at 500 nm, designated as the peak signal of I_2_. Then, we tested the UV–vis signal of the leach solution for the control film and the target film dissolved in toluene. The control film showed an I_2_ signal at 500 nm. In contrast, no I_2_ signal was observed for the target film after soaking for 24 h. This result implies that less I ion migration occurred in the target film while significant I ion migration occurred in the control perovskite film. It has been reported that the ion migration activation energy (*E*_a_) obtained from Arrhenius fitting of the conductivity of perovskite films can evaluate the ion migration behaviors in thin films.^[^ ^52^, ^57^, ^58^ ^]^ As shown in Figure 3d, the *E*_a_ of the control device is 0.24 eV while that of the target device is as high as 0.47 eV, and the larger migration energy barrier means that ion migration is more difficult to occur. This result can not only be attributed to the chemical interaction between DBzFe and perovskite film but also, as discussed earlier, the release of micro‐stress can increase the ion migration energy, thereby inhibiting ion migration. Furthermore, we conducted nudged‐elastic‐band (NEB) calculations to simulate the ion migration pathway in the unit cell and determine the energy required for migration. We focused on iodide ion transport as it was reported to have the lowest activation energy,^[^ ^59^, ^60^ ^]^ making it the most susceptible to migration/diffusion. Figure S10a and S10b (Supporting Information) illustrate overlays of 5 different structures corresponding to the 5 steps in Figure S10c (Supporting Information), defining the initial and final positions of the iodide ion during migration. For the control device, the energy barrier required for iodide ion migration from one site to another is 0.08 eV. However, when DBzFe is introduced, the energy barrier increases to 0.18 eV. This heightened barrier is likely due to the chemical interaction between DBzFe and the perovskite. Therefore, through the theoretical and experimental results, we can reasonably conclude that DBzFe can effectively suppress the ion migration in the perovskite thin film, thereby expecting to improve the stability of the device.

**Figure 4:**
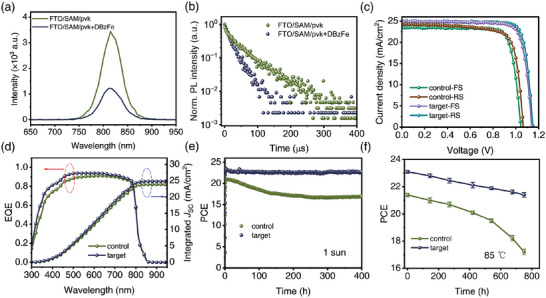
a) PL and b) TRPL of the perovskite film deposited on FTO/SAM. c) *J–V* curves of the best‐performing control and DBzFe‐modified inverted PSCs. d) IPCE curves and integrated current densities. Stability measurement of the unencapsulated devices aged under e) 1sun illumination and f) 85 °C in N_2_.

Subsequently, we studied the carrier recombination of the control and modified PSCs. PL and TRPL with the FTO/SAMs/perovskite structure with or without DBzFe modification were used to study the carrier extraction and transport. As shown in **Figure** **4**a,b, the PL intensity of the control film is lower than the target film. Meanwhile, the target film also shows a short carrier lifetime, meaning that the carrier transfer improved after introducing DBzFe (Table S1, Supporting Information). The built‐in potential (*V*_bi_) was obtained from Mott−Schottky plots in Figure S11 (Supporting Information). It is well known that *V*_OC_ is positively correlated with *V*_bi_. Compared with the control device (0.930 V), the PSCs treated with DBzFe show a higher *V*_bi_ of 1.085 V. This result suggests that charge separation and extraction were promoted after DBzFe was introduced. As a result of much inhibited nonradiative recombination due to effective defect passivation, the DBzFe‐modified device exhibited the lowest leakage current (Figure S12, Supporting Information).

We fabricated inverted MA‐ and Br‐free PSCs with the structures of FTO/SAMs/perovskite (FA_0.95_Cs_0.05_PbI_3_, with or without DBzFe)/C60/BCP/Ag. We systematically compared the photovoltaic performance of the devices modified by different concertation of DBzFe from 0 to 0.75 mg mL^−1^ (Figure S13, Supporting Information). As shown in Figure S13 (Supporting Information), the photovoltaic parameters of the device were improved after the introduction of DBzFe. When the concentration of DBzFe was 0.5 mg mL^−1^, the device obtained the highest PCE. Figure 4c illustrates the *J–V* curves for the best‐performing control and DBzFe‐modified PSCs (**Table** **1**). The control device got a maximum reverse‐scanned PCE of 20.73% (*J*_SC_ = 24.27 mA cm^−2^, *V*_OC_ = 1.065 V, and FF = 0.802). In contrast, the devices with DBzFe modification achieved much‐improved PCEs of 23.53% (*J*_SC_ = 24.95 mA cm^−2^, *V*_OC_ = 1.150 V, and FF = 0.820), which is the highest PCE of the MA‐free PSCs based on SAMs (Table S2, Supporting Information). To answer whether the DBzFe passivating molecule in this work is superior to common single‐active‐site molecules, we fabricated the devices modified by single‐active‐site molecules (Ferrocene) and compared the photovoltaic performance. As shown in Figure S14 (Supporting Information), the ferrocene‐modified devices exhibited increased PCE compared to the control device without treatment. Nevertheless, the DBzFe molecule was much more effective in enhancing PCE than the Ferrocene molecules. Figure 4d shows the control and target device's incident photon‐current conversion efficiency (IPCE) spectra. According to the IPCE, the integrated current density was estimated to be 23.82 mA cm^−2^ for control devices and 24.79 mA cm^−2^ for the target device, which were in excellent agreement with the *J*_SC_ from *J–V* curves. For control and target devices, the hysteresis index (HI) was calculated according to the formula of HI = (PCE_Reverse_ − PCE_Forward_)/PCE_Reverse_). For the control device, the HI is 7.8%, while for the target device is 4.0%. Reduced defect density and ameliorated interfacial charge extraction should be chiefly responsible for repressed hysteresis. Besides, suppressed ion migration due to the effective passivation of I^−^ vacancies and interaction between I and DBzFe should also be an essential reason for mitigated hysteresis.

**Table 1:** Photovoltaic parameters of the champion control and target devices were measured in reverse scan (RS) and forward scan (FS) under simulated AM 1.5G one sun illumination of 100  cm^−2^.

Devices		*J*_SC_ [mA cm^−2^]	*V*_OC_ [V]	FF	PCE [%]
Control	FS	23.45	1.043	0.782	19.12
	RS	24.27	1.065	0.802	20.73
Target	FS	24.77	1.139	0.801	22.60
	RS	24.95	1.150	0.820	23.53

Finally, we evaluated the stability of the control and target cells for suitability for commercialization. First, we evaluated the N_2_ stability of the unpackaged device. As shown in the figure, after 1000 h of aging, the efficiency of the control device was reduced by 9.6%, while that of the target device was decreased only by 1.5%, showing excellent N_2_ stability (Figure S15, Supporting Information). Next, we placed the unencapsulated device under 1 sun illumination to evaluate the light stability of the device. As shown in Figure 4e, after 400 h of continuous tracking, the target device still retains 96.4% of the original efficiency, while the control device only retains 80.1% of the initial efficiency, which means that DBzFe is beneficial to improve the light stability of the device. Finally, the thermal stability of the device was tested at 85 °C. As shown in Figure 4f, after 750 h of thermal aging, the control device can only retain 20% of the original efficiency, while the target device can still retain 73.5% of the initial efficiency. The improved stability can be attributed to reduced film defects, enhanced film quality, and suppressed ion migration.

## 3 Conclusion

In summary, we used a ferrocene derivative named DBzFe as an additive. The experimental results show that the perovskite film quality is significantly improved, and the defect density is reduced after the introduction of DBzFe. At the same time, the introduction of DBzFe also reduces the nonradiative recombination in the PSCs. Meanwhile, the stress in the film is released, and ion migration is suppressed. Theoretical calculation results show a chemical interaction between DBzFe and perovskite, which reduces defects and suppresses ion migration in the perovskite film. Finally, the DBzFe‐based device exhibits a high PCE of 23.5%, the highest PCE of the PSCs based on SAMs. The unencapsulated device exhibited excellent stability in the N2, light, and 85 °C environment. This finding guides the commercialization of PSCs.

## 4 Experimental Section

### Materials

All chemical reagents were used as received without further purification. All chemicals and solvents were used as received without further purification. *N, N‐*dimethylformamide (DMF, 99.8%), dimethyl sulfoxide (DMSO, 99.8%), and chlorobenzene (CB, 99.8%) were all purchased from Sigma Aldrich. Formamidium iodide (FAI, 99.9%), cesium iodide (CsI, 99.999%), and lead iodide (PbI_2_, 99.999% purity) were purchased from Advanced Election Technology CO., Ltd. 1,1′‐Dibenzoylferrocene (DBzFe, >98%) was purchased from Tokyo Chemical Industry Co., Ltd. 3‐[3‐[4‐(diphenylamino)phenyl]−9H‐carbazol‐9‐yl]propylphosphonic acid (SAM) was provided by Prof. Grigalevicius.

### Device Fabrication

FTO was ultrasonically cleaned with pure water, isopropanol, and acetonitrile for 15 min. Then, the FTO substrate was dried and cleaned with plasma for 5 min (electronic Diener, Plasma‐surface‐technology, Germany). After cooling to room temperature, the FTO was transferred to the glove box. The hole transport layer was prepared as follows: 3‐[3‐[4‐(diphenylamino)phenyl]−9H‐carbazol‐9‐yl]propylphosphonic acid was dissolved in DMF and stirred at room temperature for 10 h to prepare 1 mM solution. Then, 100 µL SAM was dropped on the FTO substrate and spin‐coated at 2000 rpm for 30 s. The sample was heated at 100 °C for 10 min. The sample was washed with DMF by spin‐coating to remove the unbonded molecules from the FTO substrate and heated at 100 °C for 3 min. After the annealed substrate was cooled for 5 min, it was ready for use. For the control perovskite films (FA_0.95_Cs_0.05_PbI_3_), 1.4 M perovskite precursor solution was prepared by dissolving 228.4 mg FAI, 18.2 mg CsI, and 645.4 mg PbI_2_ in mixed solvents of DMF and DMSO (v/v, 4/1) and stir for 2 h. Then, use the filter to filter for later use. For target perovskite films (FA_0.95_Cs_0.05_PbI_3_ with DBzFe), 1.4 M perovskite precursor solution was prepared by dissolving 228.4 mg FAI, 18.2 mg CsI, 645.4 mg PbI_2_ and DBzFe (different concentration) in mixed solvents of DMF and DMSO (v/v, 4/1) and shake for 5 min. Then, the perovskite precursor solutions without or with DBzFe were spin‐coated on the FTO/SAM substrate at 2000 rpm for 10 s and then at 4000 rpm for 40 s. During the second spin coating step, 180 µL CB was dropped onto the perovskite film at 5 s before ending the program. The perovskite sample was annealed at 100 °C for 30 min. Then, C60 (20 nm), BCP (7 nm), and Ag (100 nm) were deposited under high vacuum conditions by thermal evaporation.

### Characterization

GIXRD patterns were acquired using a Rigaku Smartlab with Cu Kα radiation (λ = 1.5406 Å). The photoluminescence (PL) and time‐resolved photoluminescence (TRPL) were measured with Jasco FP6500 Spectrofluorometer. XPS spectra were measured by S4 JPS5 90MX (JEOL, Ltd., Japan). The solar cell performances were measured using a Keithley 4200 source meter and a solar simulator under 100 mWcm^−2^ AM 1.5G in the air condition (Bunkouki CEP‐2000SRR). The effective active area of the device was defined to be 0.1 cm^2^ by using a black metal mask. Incident photon‐to‐current conversion efficiency (IPCE) spectra were recorded using a monochromatic Xenon lamp (Bunkouki CEP‐2000SRR). Mott‐Schottky (MS) and temperature‐dependent *c–f* curves were carried out via PAIOS, and the results were fitted using the companion software of PAIOS. AFM (JEOL) was employed to observe the surface roughness of perovskite thin film. Fourier transform infrared (FTIR) spectra were performed using a Thermo Scientific Nicolet 6700 FTIR spectrometer.

## Conflict of Interest

The authors declare no conflict of interest.

## Supporting information

Supporting Information

## Data Availability

The data that support the findings of this study are available from the corresponding author upon reasonable request.
