# Supplementary material for: Ferrocene Derivatives for Improving the Efficiency and Stability of MA‐Free Perovskite Solar Cells from the Perspective of Inhibiting Ion Migration and Releasing Film Stress
Source: Adv Sci (Weinh). 2023 Oct 22;10(35):2304790. doi: 10.1002/advs.202304790 (PMC10724429; doi:10.1002/advs.202304790)
Supplement: Supplementary file 1 — Supporting Information [file ADVS-10-2304790-s001.pdf]

ADVANCED SCIENCE
Open Access

Supporting Information

for *Adv. Sci.*, DOI 10.1002/advs.202304790

Ferrocene Derivatives for Improving the Efficiency and Stability of MA-Free Perovskite Solar Cells from the Perspective of Inhibiting Ion Migration and Releasing Film Stress

Huan Bi, Jiaqi Liu, Zheng Zhang, Liang Wang, Gaurav Kapil, Yuyao Wei, Ajay Kumar Baranwal, Shahrir Razey Sahamir, Yoshitaka Sanehira, Dandan Wang, Yongge Yang, Takeshi Kitamura, Raminta Beresneviciute, Saulius Grigalevicius, Qing Shen* and Shuzi Hayase**

Supporting Information

Ferrocene derivatives for improving the efficiency and stability of MA-free perovskite solar cells from the perspective of inhibiting ion migration and releasing film stress

Huan Bi[1,2], Jiaqi Liu[1], Zheng Zhang[1], Liang Wang[1], Gaurav Kapil[1], Yuyao Wei[2], Shahrir Razey Sahamir[1], Ajay Kumar Baranwal[1], Yoshitaka Sanehira[1], Dandan Wang[2], Yongge Yang[2], Takeshi Kitamura[1], Raminta Beresneviciute[3], Saulius Grigalevicius[3], Qing Shen[1,2]*, and Shuzi Hayase[1,2]**

[1]i-Powered Energy System Research Center (i-PERC), The University of Electro-Communications, 1-5-1 Chofugaoka, Chofu, Tokyo, 182-8585, Japan.

[2]Faculty of Informatics and Engineering, The University of Electro-Communications, 1-5-1 Chofugaoka, Chofu, Tokyo, 182-8585, Japan.

[3]Department of Polymers Chemistry and Technology, Kaunas University of Technology, Radvilenu Plentas 19, Kaunas, LT50254, Lithuania.

Corresponding Authors:

E-mail:

H. Bi: hbi.trans.sci@gmail.com;

Q. Shen: shen@pc.uec.ac.jp;

S. Hayase: hayase@uec.ac.jp.

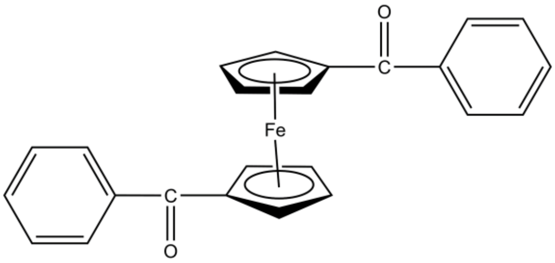

Figure S1. Structure of the DBzFe.

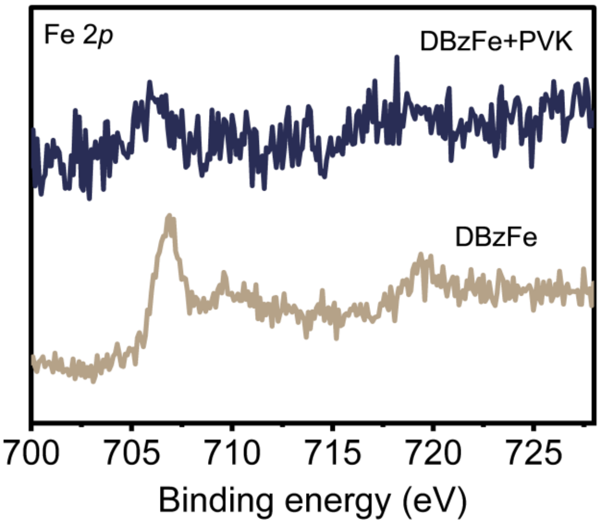

Figure S2. Fe 2*p* of the perovskite film with DBzFe modification and DBzFe powder.

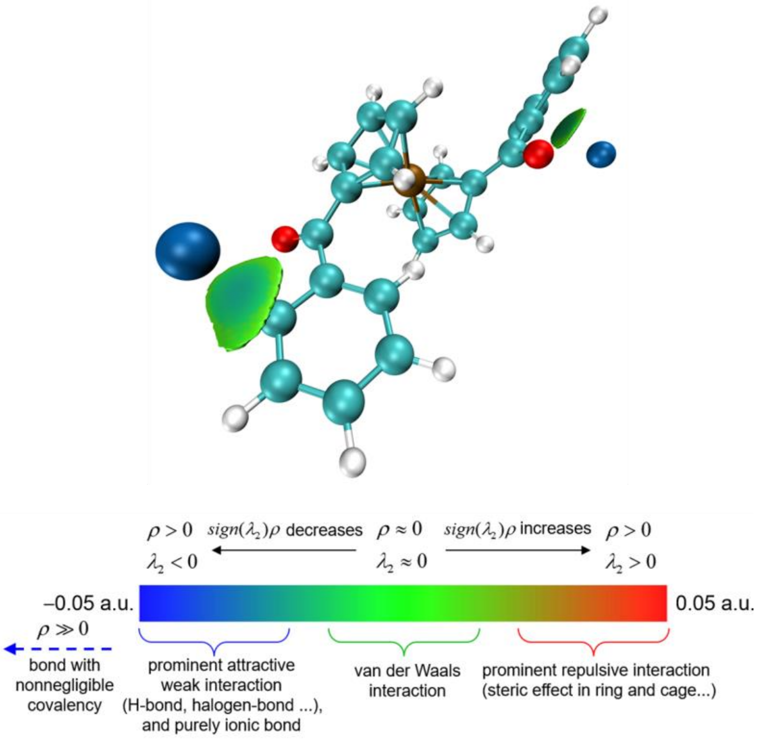

Figure S3. Sign(λ_2)ρ colored isosurfaces of δ_g^{inter} = 0.005 a.u. of DBzFe corresponding to IGMH analyse. The bottom is the common interpretation of the coloring method of the mapped function Sign(λ_2)ρ in the IGMH map.

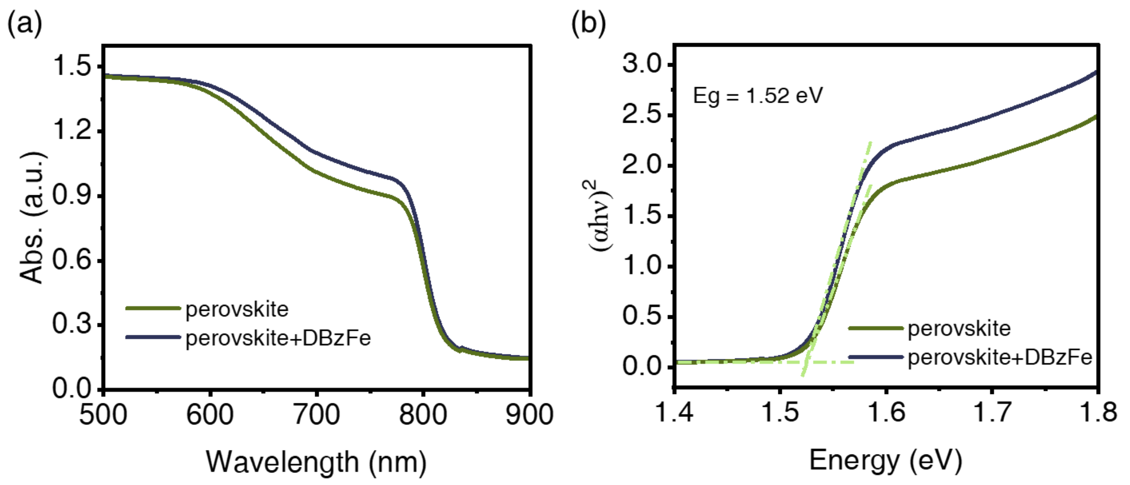

Figure S4. (a) UV-vis and (b) Tauc plot for the perovskite film with or without DbzFe modification.

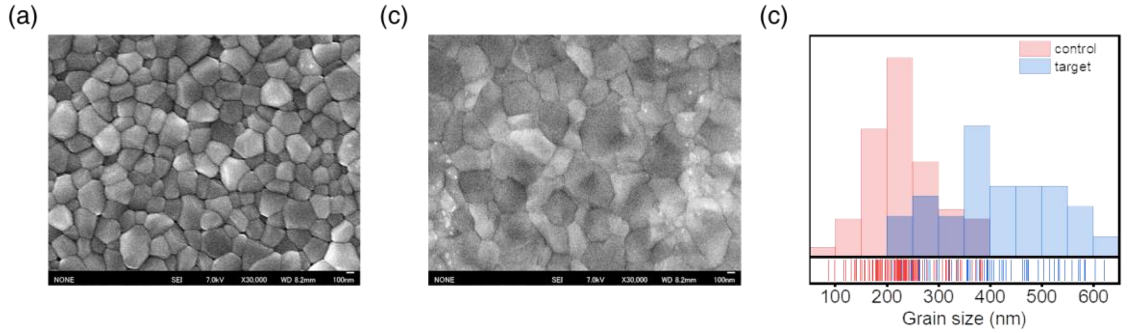

Figure S5. SEM images of the perovskite thin film are shown for (a) without the DBzFe additive and (b) with the DBzFe additive. Additionally, the grain size statistics results for the corresponding perovskite films in (a) and (b) are presented in (c).

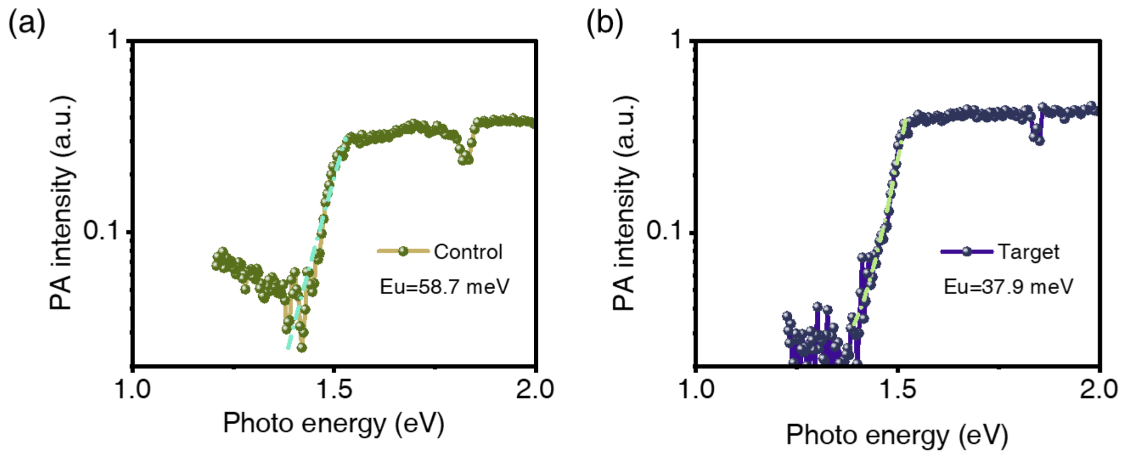

Figure S6. Optical absorption spectra of the perovskite with or without DbzFe modification.

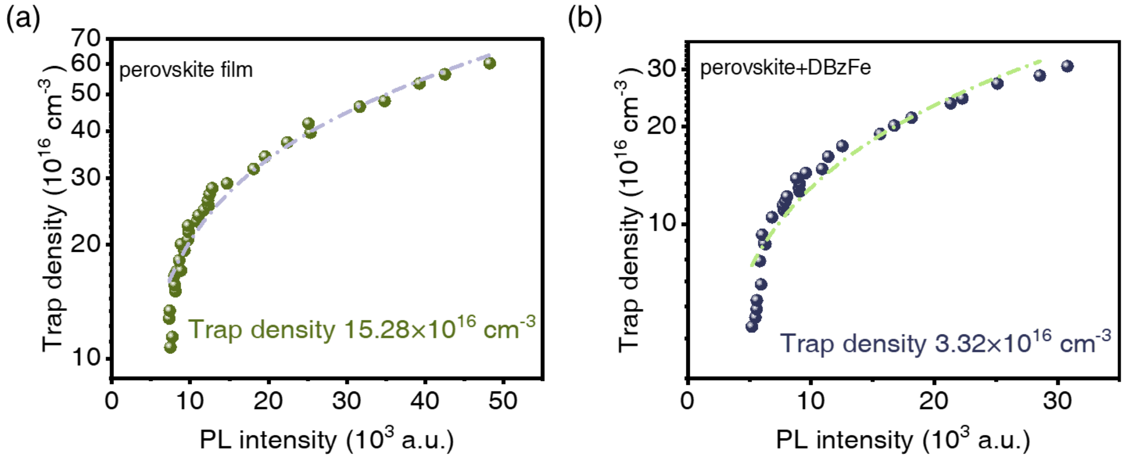

Figure S7. Photoexcited carrier density as a function of PL intensity within the low pump fluence range.

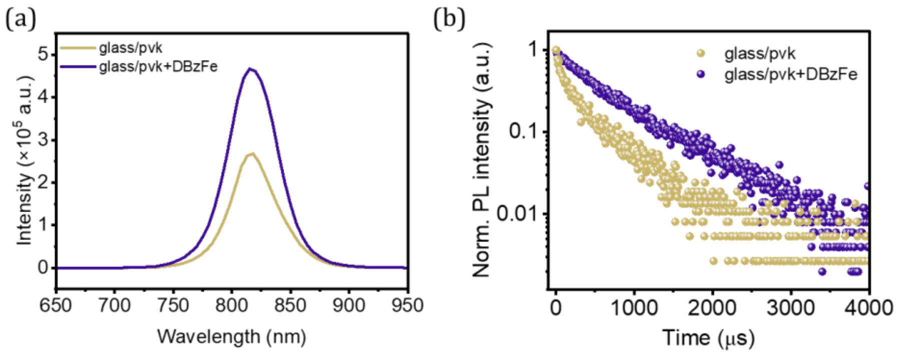

Figure S8. The (a) PL and (b) TRPL result of the perovskite film with or without DbzFe modification deposited on glass.

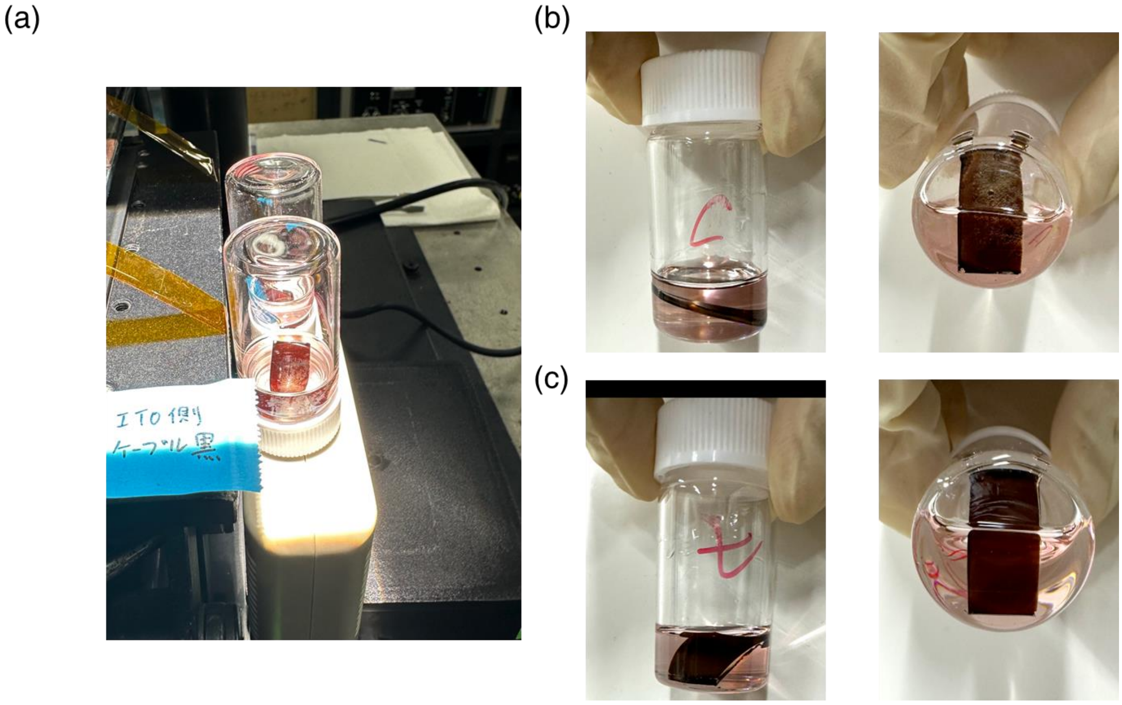

Figure S9. (a) Ion mobility evaluation device. Photographs of (b) control and (c) target films after aging for 24 h.

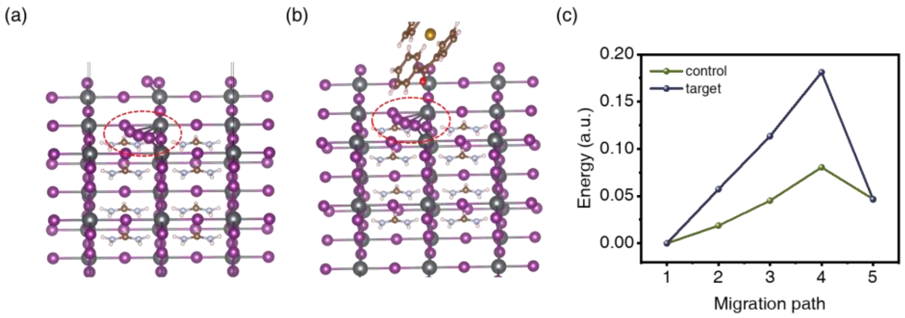

Figure S10. Migration path of I^- ions along the I^-–I^- edge of the PbI_6^{4-} octahedron in the perovskite crystal calculated from DFT method (a) without and (b) with DbzFe modifacation. (c) Iodide ion migration activation energy for $FAPbI_3$ and $FAPbI_3$ with DBzFe calculated using DFT.

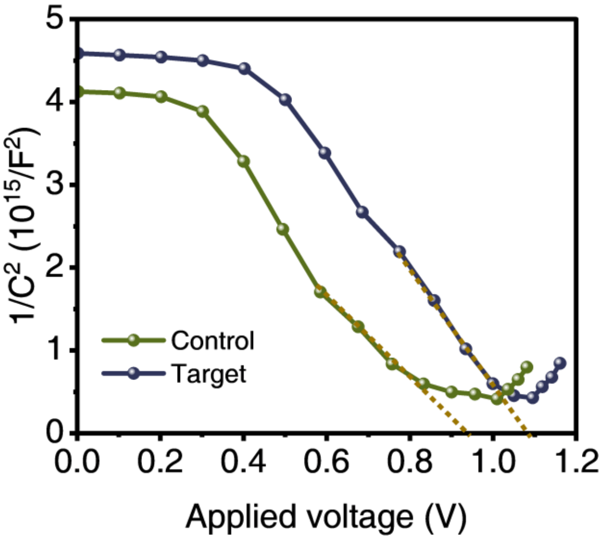

Figure S11. $1/C^2$ as a function of the applied voltage for the control and target devices. The voltage intercept of $1/C^2$ curves determined V_{bi}.

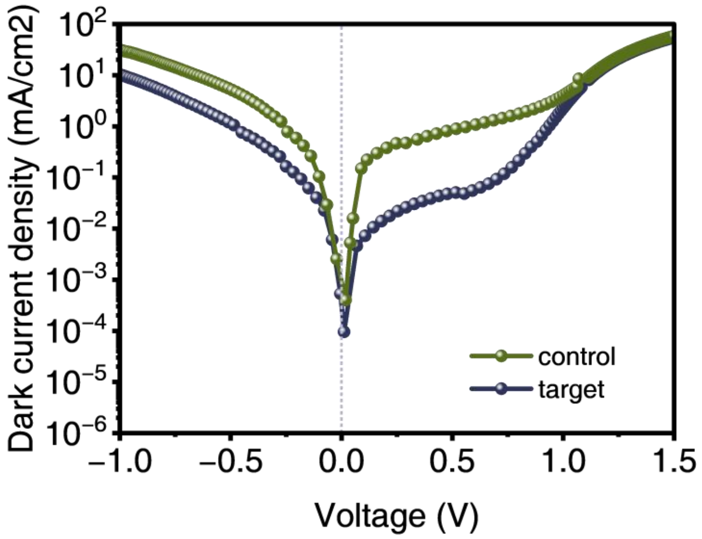

Figure S12. Dark *I*-*V* curves of the device.

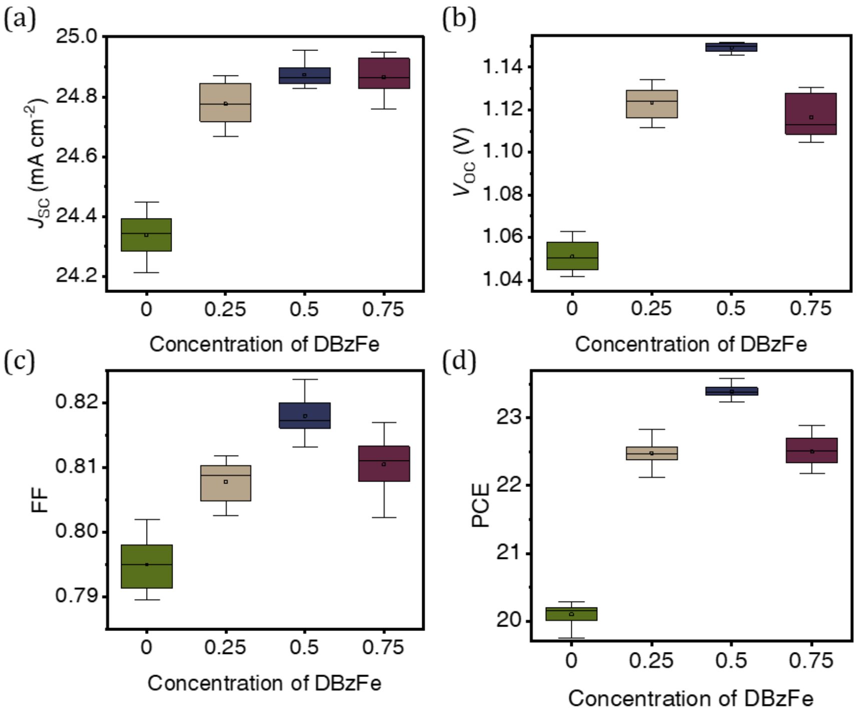

Figure S13. (a) J_{SC}, (b) V_{OC}, (c) FF, and (d) PCE statistical diagrams of the devices modified by different concentrations of DBzFe.

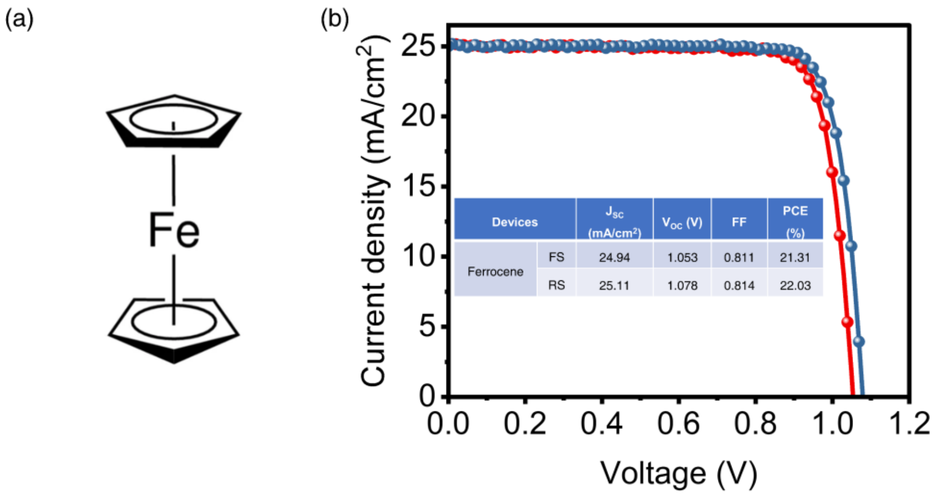

Figure S14. (a) Chemical structures of the Ferrocene. (b) *J-V* curves of the devices based on Ferrocene. The inset shows the photovoltaic parameters of the device based on Ferrocene.

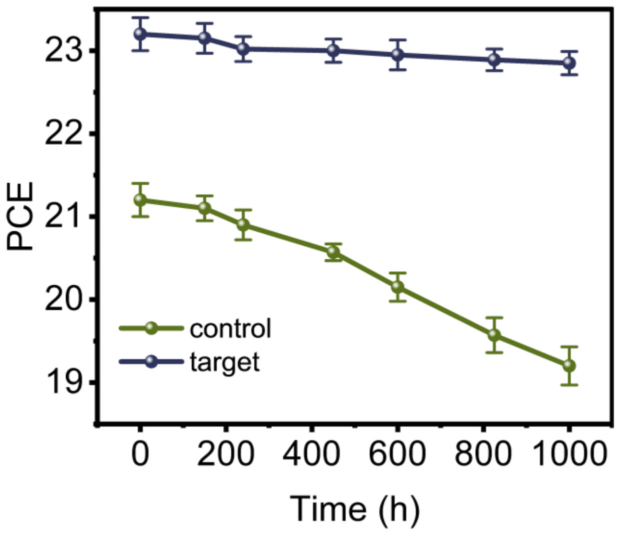

Figure S15. Stability measurement of the unencapsulated devices aged in N_2.

Table S1. Fitted results from TRPL in Figure 4b.

Devices	τ_1 (ns)	A_1	τ_2 (ns)	A_2	τ_{ave} (ns)
FTO/SAM/pvk	2.49	0.38	43.25	0.62	41.87
FTO/SAM/pvk+DBzFe	3.96	0.45	26.05	0.54	23.57

Table S1. Summary of photovoltaic performance of reported high-efficiency SAMs-based PSCs to date. For comparison, the photovoltaic parameters of our best-performing device were incorporated in this table.

Device structure	J_{SC} (mA/cm^2)	V_{OC} (V)	FF	PCE (%)	Ref.
**FTO/SAM/$FA_{0.95}Cs_{0.05}PbI_3$+DBzFe/C60/BCP/Ag**	**24.95**	**1.150**	**0.820**	**23.53**	**This work**
ITO/V1036/C4/$Cs_{0.05}(MA_{0.17}FA_{0.83})_{0.95}Pb(I_{0.83}Br_{0.17})_3$/C60/BCP/Cu	21.9	1.09	0.810	17.8	1
ITO/MeO–2PACz/$Cs_{0.05}(MA_{0.17}FA_{0.83})_{0.95}Pb(I_{0.83}Br_{0.17})_3$/C60/BCP/Cu	22.2	1.144	0.805	20.4	2
ITO/BCB-C4PA/$Cs_{0.07}FA_{0.9}MA_{0.03}Pb(I_{0.92}Br_{0.08})_3$/C60/BCP/Ag	24.4	1.13	0.800	22.2	3

ITO/2PACz/$Cs_{0.05}(MA_{0.17}FA_{0.83})_{0.95}Pb(I_{0.83}Br_{0.17})_3$/$C_{60}$/BCP/Cu	21.9	1.188	0.802	20.9	2
ITO/MC-43/$Cs_{0.05}(MA_{0.17}FA_{0.83})_{0.95}Pb(I_{0.83}Br_{0.17})_3$/PCBM/Ag	20.3	1.07	80.0	17.3	4
ITO/TPA/$Cs_{0.05}(MA_{0.17}FA_{0.83})_{0.95}Pb(I_{0.83}Br_{0.17})_3$/PCBM/Ag	19.4	1.06	76.9	15.9	4
ITO/EADR03/$Cs_{0.05}FA_{0.79}MA_{0.16}Pb(I_{0.84}Br_{0.16})_3$/LiF/$C_{60}$/BCP/NaF/Cu	22.9	1.156	80.0	21.2	5
ITO/TPT-P6/$Cs_{0.05}MA_{0.12}FA_{0.83}Pb(I_{0.85}Br_{0.15})_3$/$C_{60}$/BCP/Ag	23.50	1.125	81.08	21.43	6
ITO/Br–2EPT/$Cs_{0.05}(FA_{0.92}MA_{0.08})_{0.95}Pb(I_{0.92}Br_{0.08})_3$/$C_{60}$/BCP/Cu	25.11	1.09	82.0	22.44	7
ITO/TPA-PTC6/CABr/$MAPbI_3$/PCBM/BCP/Ag	21.8	1.039	77.35	17.49	8
ITO/MeO–2PACz/$FASnI_3$/C_{60}/BCP/Ag	20.3	0.475	67.3	6.49	9
ITO/EA-58/$MAPbI_3$/PCBM/Ca/Ag	19.08	0.967	76.37	13.71	10

Note S1 Trap States Density Determination.

The dynamics of photogenerated charge carrier density can relax through band-edge emission or trapassisted nonradiative routes in the low fluence excitation zone, where Auger recombination is insignificant. The initial charge carrier density injected by optical pumping at the film surface was estimated by multiplying the laser pulse photon fluence times the film absorption coefficient, and the defect density can then be calculated by fitting the following equation:

$$n_c = \sum_i n_{TP}^i \left(1 - e^{\frac{a_i \tau_0 I_{PL}}{k}}\right) + \frac{I_{PL}}{k}$$

where n_{TP}^i is the initial trap states density, a_i is the product of trapping cross section

and carrier velocity, τ_0 is the PL decay lifetime, and k is a constant. It was in the range from 10^{15} to 10^{18} cm^{-3}, for laser pulse fluence varied from 10^{-9} J/cm^2 to 2×10^{-7} J/cm^2 (473 nm, 10 ps, 8 MHz).

References

(1) Magomedov A.; Al-Ashouri A.; Kasparavičius E.; Strazdaite S.; Niaura G.; Jošt M.; Malinauskas T.; Albrecht S.; Getautis V., Self-Assembled Hole Transporting Monolayer for Highly Efficient Perovskite Solar Cells. *Adv. Energy Mater.* **2018**, *8*, 1801892.

(2) Al-Ashouri A.; Magomedov A.; Roß M.; Jošt M.; Talaikis M.; Chistiakova G.; Bertram T.; Márquez J. A.; Köhnen E.; Kasparavičius E.; Levcenco S.; Gil-Escrig L.; Hages C. J.; Schlatmann R.; Rech B.; Malinauskas T.; Unold T.; Kaufmann C. A.; Korte L.; Niaura G.; Getautis V.; Albrecht S., Conformal monolayer contacts with lossless interfaces for perovskite single junction and monolithic tandem solar cells. *Energy Environ. Sci.* **2019**, *12*, 3356-3369.

(3) Wang W.; Wei K.; Yang L.; Deng J.; Zhang J.; Tang W., Dynamic self-assembly of small molecules enables the spontaneous fabrication of hole conductors at perovskite/electrode interfaces for over 22% stable inverted perovskite solar cells. *Materials Horizons* **2023**.

(4) Yalcin E.; Can M.; Rodriguez-Seco C.; Aktas E.; Pudi R.; Cambarau W.; Demic S.; Palomares E., Semiconductor self-assembled monolayers as selective contacts for efficient PiN perovskite solar cells. *Energy Environ. Sci.* **2019**, *12*, 230-237.

(5) Aktas E.; Phung N.; Köbler H.; González D. A.; Méndez M.; Kafedjiska I.; Turren-Cruz S.-H.; Wenisch R.; Lauermann I.; Abate A.; Palomares E., Understanding the perovskite/self-assembled selective contact interface for ultra-stable and highly efficient p–i–n perovskite solar cells. *Energy Environ. Sci.* **2021**, *14*, 3976-3985.

(6) Wang J.; Liu W.; Luo G.; Li Z.; Zhao C.; Zhang H.; Zhu M.; Xu Q.; Wang X.; Zhao C.; Qu Y.;

Yang Z.; Yao T.; Li Y.; Lin Y.; Wu Y.; Li Y., Synergistic effect of well-defined dual sites boosting the oxygen reduction reaction. *Energy Environ. Sci.* **2018**, *11*, 3375-3379.

(7) Ullah A.; Park K. H.; Nguyen H. D.; Siddique Y.; Shah S. F. A.; Tran H.; Park S.; Lee S. I.; Lee K.-K.; Han C.-H.; Kim K.; Ahn S.; Jeong I.; Park Y. S.; Hong S., Novel Phenothiazine-Based Self-Assembled Monolayer as a Hole Selective Contact for Highly Efficient and Stable p-i-n Perovskite Solar Cells. *Adv. Energy Mater.* **2022**, *12*, 2103175.

(8) Zhang H.; Wu Y.; Zhang W.; Li E.; Shen C.; Jiang H.; Tian H.; Zhu W.-H., Low cost and stable quinoxaline-based hole-transporting materials with a D–A–D molecular configuration for efficient perovskite solar cells. *Chem. Sci.* **2018**, *9*, 5919-5928.

(9) Song D.; Narra S.; Li M.-Y.; Lin J.-S.; Diau E. W.-G., Interfacial Engineering with a Hole-Selective Self-Assembled Monolayer for Tin Perovskite Solar Cells via a Two-Step Fabrication. *ACS Energy Lett.* **2021**, *6*, 4179-4186.

(10) Akın Kara D.; Kara K.; Oylumluoglu G.; Yigit M. Z.; Can M.; Kim J. J.; Burnett E. K.; Gonzalez Arellano D. L.; Buyukcelebi S.; Ozel F.; Usluer O.; Briseno A. L.; Kus M., Enhanced Device Efficiency and Long-Term Stability via Boronic Acid-Based Self-Assembled Monolayer Modification of Indium Tin Oxide in a Planar Perovskite Solar Cell. *ACS Appl. Mater. Interfaces* **2018**, *10*, 30000-30007.
